# Autologous adipose mesenchymal stem cell administration in arteriosclerosis and potential for anti-aging application: a retrospective cohort study

**DOI:** 10.1186/s13287-020-02067-x

**Published:** 2020-12-11

**Authors:** Hiroki Ohta, Xiaolan Liu, Miho Maeda

**Affiliations:** Regenerative Medicine, Sun Field Clinic, TIME24 Building 1F 2-4-32 Aomi, Koto-ku, Tokyo, 135-0064 Japan

**Keywords:** Adipose-derived mesenchymal stem cell, Anti-aging, Arteriosclerosis, Safety, Efficacy

## Abstract

**Objective:**

Arteriosclerosis is an age-related disease and a leading cause of cardiovascular disease. In animal experiments, mesenchymal stem cells and its culture-conditioned medium have been shown to be promising tools for prevention or treatment of arteriosclerosis. On the basis of these evidences, we aimed to assess whether administration of autologous adipose-derived mesenchymal stem cells (Ad-MSC) is safe and effective for treatment of arteriosclerosis.

**Methods:**

We retrospectively reviewed clinical records of patients with arteriosclerosis who had received autologous Ad-MSC administration at our clinic. Patients’ characteristics were recorded and data on lipid profile, intimal-media thickness (IMT), cardio-ankle vascular index (CAVI), and ankle-brachial index (ABI) before and after Ad-MSC administration were collected and compared.

**Results:**

Treatment with Ad-MSC significantly improved HDL, LDL, and remnant-like particle (RLP) cholesterol levels. No adverse effect or toxicity was observed in relation to the treatment. Of the patients with abnormal HDL values before treatment, the vast majority showed improvement in the values. Overall, the measurements after treatment were significantly increased compared with those before treatment (*p* < 0.01). In addition, decreases in LDL cholesterol and RLP levels were observed after treatment in patients who had abnormal LDL cholesterol or RLP levels before treatment. The majority of patients with pre-treatment abnormal CAVI values had improved values after treatment. In patients with available IMT values, a significant decrease in the IMT values was found after therapy (*p* < 0.01). All patients with borderline arteriosclerosis disease had improved laboratory findings after treatment. In general, post-treatment values were significantly decreased as compared with pre-treatment values. Of the patients with normal ABI values before treatment at the same time as CAVI, the vast majority remained normal after treatment.

**Conclusions:**

These findings suggest that Ad-MSC administration is safe and effective in patients developing arteriosclerosis, thereby providing an attractive tool for anti-aging application.

## Background

Arteriosclerosis is an age-related disease and is considered a leading cause of death and disability in adults worldwide [1]. Minimally invasive treatment is developing with many endovascular treatments being indicated for vascular disease [2]. The pitfalls in the study of vascular disease are also becoming more apparent. However, there is still room for improvement [3]. Arteriosclerosis develops as a slow and progressive disease of blood vessels during the aging process mainly affecting large (aorta, pulmonary, and iliac arteries) and medium size arteries (coronary, cerebral, and limbs arteries) leading to cardiovascular disease. In general, blood vessels lose some degree of elasticity (loss of elasticity) with aging and subsequently a build-up of fatty deposits (plaque) in the wall of an artery begins [4]. Contributory factors such as smoking, hypertension, diabetes mellitus, obesity, hyperlipidemia, and family history will promote the disease [5].

Pathology of arteriosclerosis comprises hardening of the arterial wall and narrowing of the lumen caused by gradual build-up of plaque in the arterial wall. Plaque formation restricts blood flow lumen and contributes to the development of coronary artery disease. Moreover, plaque rupture and thrombus formation lead to many ischemic cardiac events. Arteriosclerosis is considered a modern lifestyle disease [6, 7] and its incidence is decreasing due to better understanding and change in life style. The diagnosis of arteriosclerosis includes physical examination and laboratory tests and various imaging methods. One convenient method is to assess carotid artery intima-media thickness (IMT). IMT has been identified as a risk factor for myocardial infarction and stroke in older adults [8], and in diabetes mellitus, intensive diabetes treatment resulted in decreased progression of intima-media thickness 6 years after the end of the trial [9]. Other available assessment tests include cardio-ankle vascular index (CAVI) and ankle-brachial index (ABI). CAVI is a well-known indicator of arterial stiffness from the origin of the aorta to the ankle and is theoretically independent of blood pressure at the time of measurement. CAVI increases in parallel with age and is elevated even in mild arteriosclerotic disease. On the other hand, ABI is the ratio of the blood pressure at the ankle to the blood pressure in the arm [10]. CAVI can be measured simultaneously with the measurement of ABIs, and it is a test method with little burden for patients [11]. CAVI and ABI are widely used because of the ease of examination.

Several strategies are available to treat and prevent arteriosclerosis including dietary regimens, changes in the lifestyle, and cholesterol lowering drugs. The most frequent drug therapy for this disease includes statins which are associated with some adverse effects. Recently, with the development of stem cell technology, new therapeutic approaches have become available for various diseases including arteriosclerosis. Among stem cell therapies, human mesenchymal stem cells (MSC) and particularly adipose-derived mesenchymal stem cells (Ad-MSC) have attracted much attention due to their extremely low or no adverse reactions [12–15]. Long-term follow-up of patients with ischemic stroke after MSC administration showed some improvement with no adverse effects after 5 years [16]. In addition, conditioned medium (CM) derived from the culture of such stem cells has also been collected and used for therapy [15, 17]. In general, Ad-MSC possess pluripotential properties capable of differentiating into several types of cells such as adipocyte, myocyte, chondrocyte, and osteocyte [16]. In a rodent study, MSC prevented inflammatory and apoptotic processes involved in brain injury by suppressing pro-inflammatory cytokines [18, 19]. It inhibited expression of cytokines such as IL-1α, IL-1β, IL-6, and TNF-α in the CSF and periventricular brain region. It also modulated immune function by impairing the differentiation of dendritic cells that are the main antigen-presenting cells in human immunity [20]. Taken together, these results suggested the importance of immunomodulatory function of MSC which can also be applicable to the effect of Ad-MSC.

Ischemic heart disease and cerebrovascular disease caused by arteriosclerosis not only are life-threatening, but also inflict various complications after treatment such as impaired motor skills and cognitive dysfunction. As a result, the quality of life (QOL) and activities of daily living (ADL) will be reduced, and long-term costly medical care will be enforced. Inflammatory processes play a major role in the initiation and progression of arteriosclerosis and Ad-MSC possesses strong anti-inflammatory and immunomodulatory capacity and thus may provide a potential therapeutic tool to prevent or treat arteriosclerosis. Here, we retrospectively studied a cohort of patients with arteriosclerosis who had received autologous Ad-MSC administration at our clinic. We focused on the safety and efficacy of this approach as an adjunct to the standard treatment modalities for this disease. To our knowledge, this is the first report evaluating the potential application of Ad-MSC in patients with arteriosclerosis.

## Methods

### Study design and patients

This retrospective study was performed following the ethical policies of the Sun Field Clinic in Tokyo, Japan, and were approved by its related Ethics Committee of the World Academy of Anti-Aging and Regenerative Medicine (approval No. 4-001). In addition, written informed consent and consent to publish had been obtained from the patients before performing the procedures. Inclusion and exclusion criteria were as follows:

*Inclusion criteria*. (1) Patients aged 18 years or older; (2) patients who were judged to have arteriosclerotic lesions and who had two or more relatives within the second degree with arteriosclerotic disease; (3) patients who were judged to have a high predisposition to arteriosclerosis as a result of genetic testing or who met the following selection criteria and have no conflicts with exclusion criteria: (a) pulse wave velocity (PWV) ≥ 1400 (cm/s) or cardio-ankle vascular index (CAVI) ≥ 8.0, (b) ankle-brachial index (ABI) ≤ 0.9, and (c) carotid ultrasonography with IMT ≥ 1.1 in the carotid arteries (common carotid artery, internal carotid artery); (4) patients who remained fit and healthy enough to allow fat tissue collection; (5) patients who have the normal ability to give informed consent or who have obtained informed consent from their legally acceptable guardian; (6) patients who have been given sufficient explanation and a consent form for this treatment and have given their voluntary informed consent (if the patient does not have the ability to consent, then their legal guardian had to agree in writing); and (7) patients who were deemed eligible by the physician in charge through interview, examinations, and else.

*Exclusion criteria*. We excluded patients having any of the following infections using virus and bacterial tests: (1) HIV (antigen antibody method), (2) HCV antibody (CLIA method), (3) HBs antigen (CLIA method), (4) HBs antigen (CLIA method), (5) HTLV-I antibody (CLEIA method), (6) syphilis (RPR method, TPHA method), (7) herpes simplex (CF method), (8) mycoplasma (PA method), and (9) parvovirus B19 (IgM antibody).

We confirmed that there were no pathogen infections. In the event of an infection, the fat tissue collection and treatment were terminated.

In all patients, a blood examination had been first performed including tests for infectious diseases such as hepatitis virus, rubella virus, herpes zoster virus, and HIV, which are generally performed before surgical procedures to determine if stem cell culture will be compromised. If even one of these results was outlier, the patient was not eligible for stem cell therapy. Specifically, in addition to general blood tests, cholesterol levels, carotid ultrasonography, and ABI/CAVI, which are tests related to arteriosclerosis, were performed. For data tested multiple times, the worst values before cell administration were compared with the best values after cell administration.

### Preparation of autologous Ad-MSC

Autologous Ad-MSC had been prepared as follows and preserved in liquid nitrogen. Adipose tissue was harvested from the peri-umbilical area of the patient using a simple lipo-aspiration method under local anesthesia and used for Ad-MSC isolation. A total amount of approximately 20 g of fatty tissue was obtained. The patients had undergone rigorous laboratory examinations for viruses and other pathogens to ensure pathogen-free preparations.

The isolation and characterization of Ad-MSC followed the conventional methods described in several reports [21–24] and also reported in detail in our previous publication [15]. To avoid issues related to bovine serum protein, such as prion-related encephalopathy or other xenogeneic infections, a proprietary human and animal-origin-free (AOF) medium (BioMimetics Sympathies, Inc., and ROHTO Pharmaceutical Co., Ltd.., Tokyo, Japan) for culture and expansion of the stem cells had been used [25].

The Ad-MSC had been selected and expanded for 3 to 4 passages. After each passage, the cells and their culture supernatant medium were separated. The Ad-MSC were suspended in a cryopreservation solution at a cell concentration of 1 × 10^7^ cells/ml and immediately cryopreserved in liquid nitrogen. A mean of 16 ± 3 days was required for the production of Ad-MSC and the time needed for thawing the cryopreserved cells and making it ready for administration was less than 15 min.

To ensure safety, all cell product manufacturing procedures had complied with the principles of current GMP. Strict quality control and quality assurance measures had been applied. The quality control of the final Ad-MSC preparations had been assessed. For quality control, the Ad-MSC preparations had been checked for cell survival by trypan blue staining method, genetic stability by conventional karyotyping using G-banding method and microbiological, mycoplasma, and endotoxin contamination by nucleic acid testing-based assay and limulus amebocyte lysate kinetic turbidimetric assay. Environmental and microbiological controls had been used throughout the Ad-MSC manufacturing process.

### Administration of hAd-MSC

The Ad-MSC were administered intravenously. The number of Ad-MSC in transfusion events ranged from 2930 to 20,000 × 10^4^ cells. The Ad-MSC were injected into 250-ml ringer lactate solution bottle and mixed by gentle moving to achieve a homogenous solution without cell aggregate particles. The intravenous transfusion time took approximately 40 min and the vital signs were checked regularly during transfusion. The mean number of injections was 1.58 ± 0.95 (range 1–4). After transfusion, the patient was rested at the clinic for 1 h and vital signs were regularly checked. If no abnormality was found, the patient was discharged and advised to call the clinic the next day for check-up.

### Data collection and processing

Clinical records of patients who had received Ad-MSC treatment at our clinic were reviewed. Demographic information, height, weight, comorbid conditions, cardiovascular risk factors, a history of claudication, cardio-ankle vascular index (CAVI), ankle-to-brachial index (ABI), and a list of current medications were recorded during the medical history interview. Routine blood examination including cardiovascular, liver, and renal function tests had been performed before and/or after treatment. We analyzed data for significance of difference between values before and after therapy. The time periods of data between before and after stem cell administration varied from patient to patient.

### Statistical analysis

The data were analyzed using PRISM ver.8.4.0 (GraphPad Software, Inc., CA, USA). The significance of differences between the two groups was tested by either paired *t* test or Fisher’s exact tests as appropriate, at the 0.05 significance level.

## Results

### Patient’s characteristics

Baseline patients’ characteristics are listed in Table 1. There were 78 patients (mean age ± SD = 55 ± 12; range 20–82) who had received Ad-MSC for arteriosclerosis including 43 males (mean age 55.95 ± 10.93; range 20–73) and 35 females (mean age 52.45 ± 14.39; range 27–82). Of the 78 patients, 12 had abnormal HDL (≤ 40 mg/dL) (mean 32.8 ± 4.9; range 23–39), 8 had abnormal LDL (≥ 140 mg/dL) (mean 170.5 ± 24.9; range 142–204), and 8 had abnormal remnant-like particle (RPL) (≥ 7.5 mg/dL) (mean 15.29 ± 10.76; range 7.8–65) cholesterol values before therapy. Genetic predisposition within two pedigrees was found in 2 males and 3 females. Of all patients, 16 were smokers and 62 were non-smokers. Of the 78 patients, 29 had right-side abnormal IMT (≥ 1.1 mm) (mean 1.31 ± 0.20; range 1.1–1.7) and 26 had left-side abnormal IMT (mean 1.26 ± 0.17; range 1.1–1.7) values before and after treatment allowing a comparative analysis. Right-side abnormal CAVI (≥ 8.0) was available in 22 and left-side abnormal CAVI in 21 cases. No patient had abnormal ABI (≤ 0.9) before treatment.

**Table 1:** Baseline characteristics of patients

Variable	No. of cases	Mean ± SD (range)
Sex		
Male	43	NA
Female	35	NA
Age (years)		
Male	43	55.95 ± 10.93 (20–73)
Female	35	52.45 ± 14.39 (27–82)
Smoking habit		
Smoker	16	NA
Non-smoker	62	NA
Abnormal LDL (≥ 140 mg/dL)	8	170.5 ± 24.9 (142–204)
Abnormal RLP (≥ 7.5 mg/dL)	28	15.29 ± 10.76 (7.8–65)
IMT (≥ 1.1 mm)		
Abnormal Rt side	18	1.31 ± 0.20 (1.1–1.7)
Abnormal Lt side	18	1.26 ± 0.17 (1.1–1.7)
CAVI (8.0–10.8)		
Abnormal Rt side	12	9.95 ± 0.85 (9–12)
Abnormal Lt side	11	9.98 ± 1.56 (9–13.6)

### Administration of Ad-MSC

**Fig. 1:**
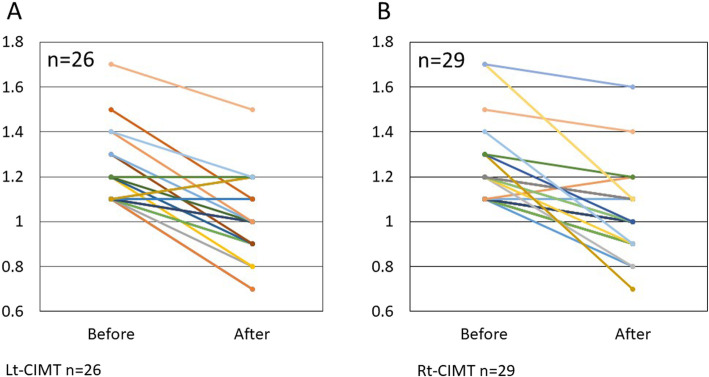
In the 12 patients with abnormal HDL values (≤ 40 mg/dL) before treatment, 11 of 12 (91.7%) showed improvement in the values (**a**). In addition, of the 8 patients who had abnormal LDL levels before treatment, 6 (75%) revealed decreases in LDL cholesterol levels after treatment (**b**). This was also true in the vast majority of cases with abnormal RLP levels, 24 out of 28 cases (85.7%) (**c**)

Treatment with Ad-MSC significantly improved HDL, LDL, and remnant-like particle (RLP) cholesterol levels (Table 2). No adverse effect or toxicity was observed in direct relation to the treatment. Of 12 patients with abnormal HDL values before treatment, 11 (91.7%) showed improvement in the values (Fig. 1a). Overall, measurements after treatment were significantly increased compared with those before treatment (*p* < 0.01). In addition, of the 8 patients who had abnormal LDL levels before treatment, 6 (75%) revealed decreases in LDL cholesterol levels after treatment (Fig. 1b). This was also true in the vast majority of cases with abnormal RLP levels, 24 out of 28 cases (85.7%) (Fig. 1c).

**Table 2:** Clinical and laboratory data

Variable	No. of cases	Before	Age	*p* value
		Mean ± SD mg/dl (range)		
Abnormal HDL (≤ 40 mg/dL)	12	32.8 ± 4.9 (23–39)	36.2 ± 5.0 (33–49)	< 0.01
Abnormal LDL (≥ 140 mg/dL)	8	170.5 ± 24.9 (142–204)	144.5 ± 15.56 (138–175)	< 0.01
Abnormal RLP (≥ 7.5 mg/dL)	28	15.29 ± 10.76 (7.8–65)	9.06 ± 6.62 (3.3–34.9)	< 0.01
IMT (≥ 1.1 mm)				
Abnormal Rt side	18	1.31 ± 0.20 (1.1–1.7)	1.07 ± 0.20 (0.7–1.6)	< 0.01
Abnormal Lt side	18	1.26 ± 0.17 (1.1–1.7)	1.06 ± 0.18 (0.7–1.5)	< 0.01
CAVI (8.0–10.8)				
Abnormal Rt side	12	9.95 ± 0.85 (9–12)	9.4 ± 1.55 (7.7–12.8)	< 0.01
Abnormal Lt side	11	9.98 ± 1.56 (9–13.6)	9.12 ± 1.24 (7.9–10.9)	< 0.01

**Fig. 2:**
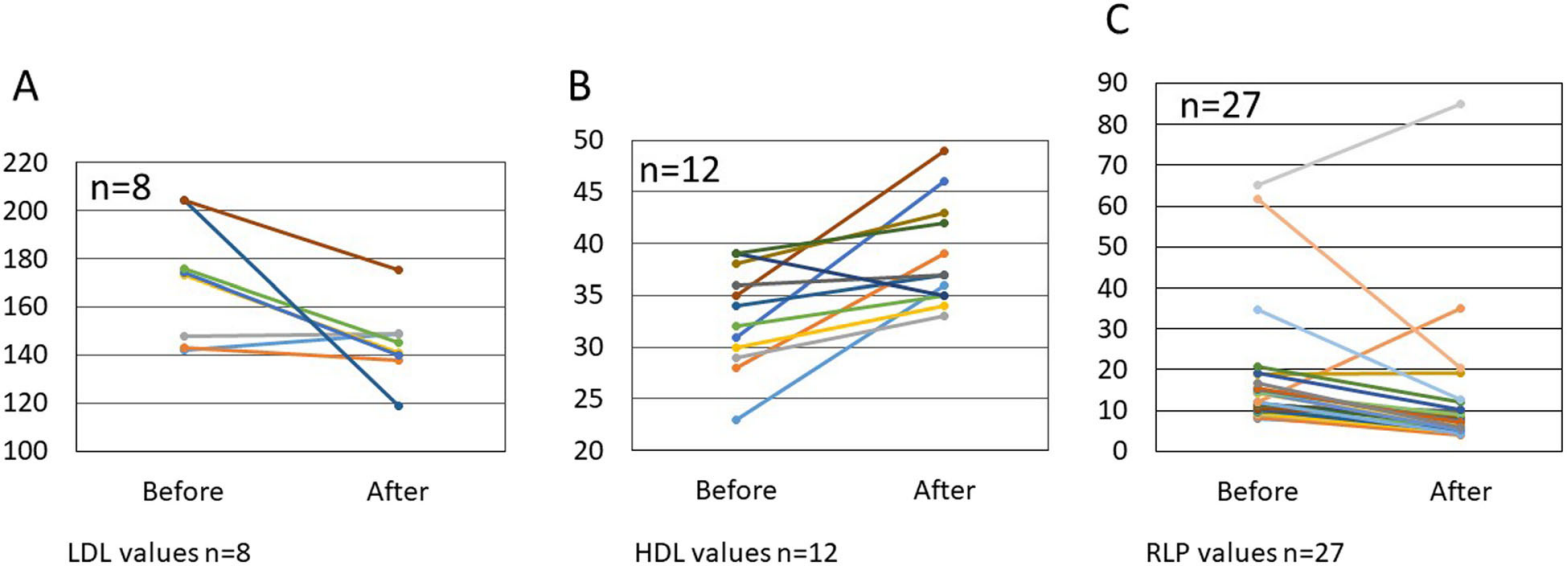
In patients with pre-treatment abnormal CAVI values (≥ 8.0), 11 of 12 (91.7%) with right-side CAVI (**a**) and 10 of 11 (90.9%) with left-side CAVI (**b**) had improved values after treatment

**Fig. 3:**
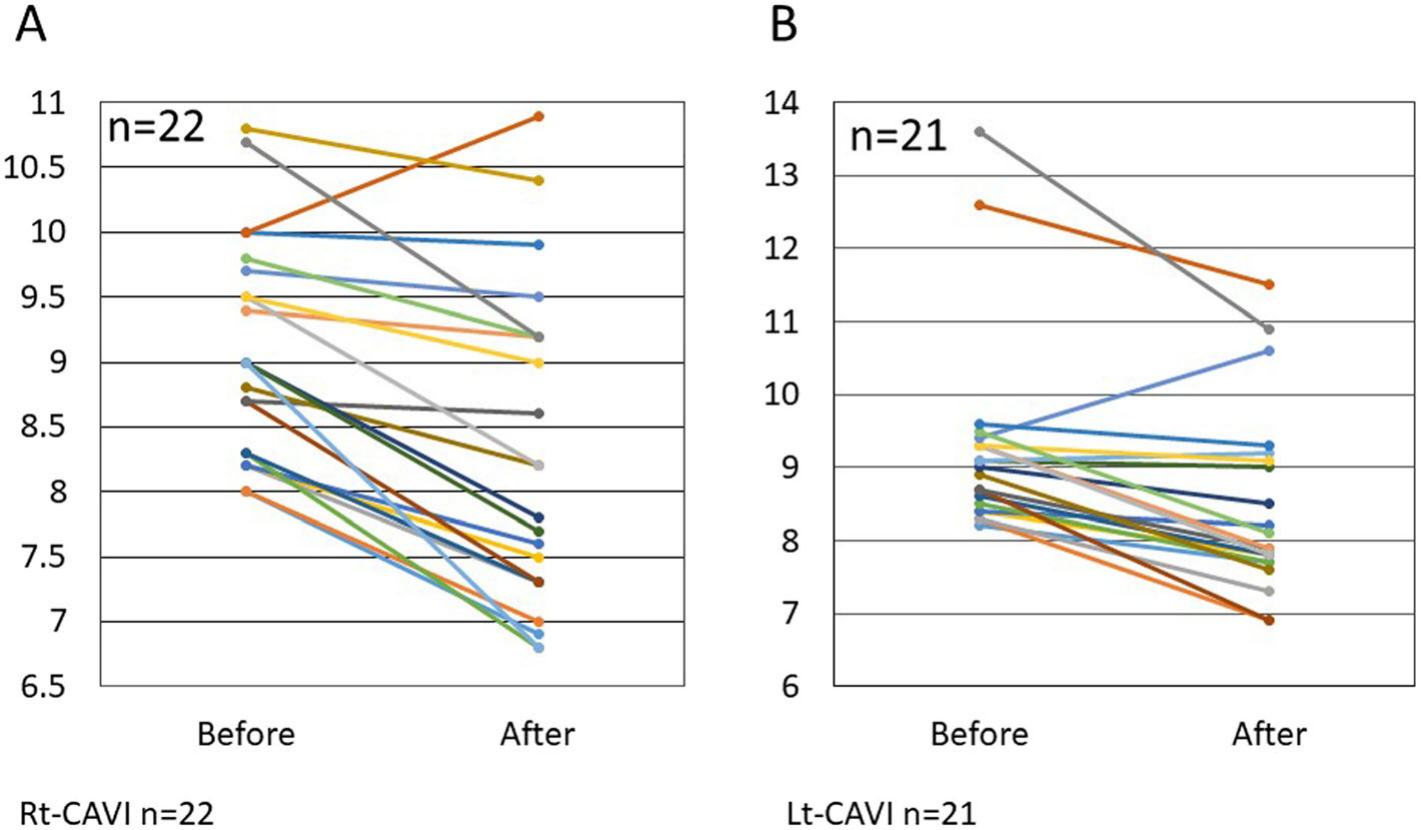
In the 18 patients with abnormal IMT (≥ 1.1 mm) values before treatment (**a**), a significant decrease in the IMT values (*P* < 0.001) was found after therapy (**b**)

**Fig. 4:**
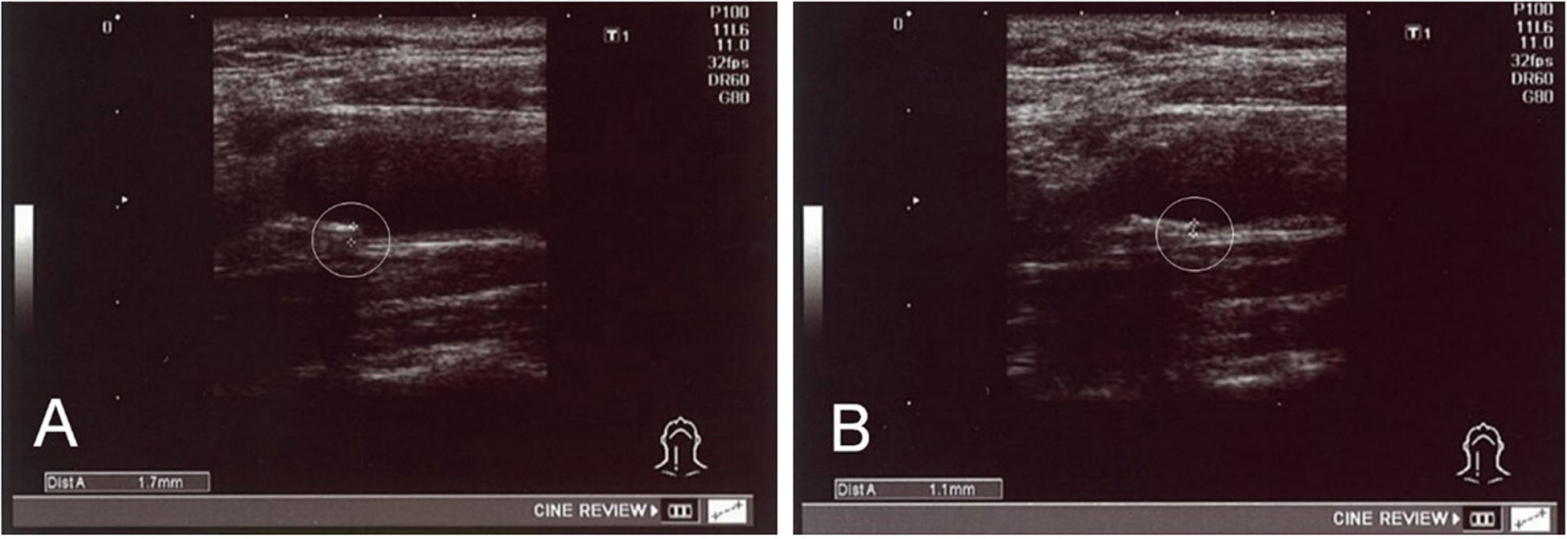
An example of common carotid artery IMT in a 56-year-old male patient before (**a**) and after (**b**) treatment with Ad-MSC shows a decrease in IMT from 1.7 to 1.1 mm nearly 4 months after therapy

In patients with pre-treatment abnormal CAVI values, 11of 12 (91.7%) and 10 of 11 (90.9%), had improved right-side and left-side CAVI values, respectively, after treatment (Fig. 2a, b). In 18 patients with abnormal IMT values before treatment (Fig. 3a), a significant decrease in the IMT values (*p* < 0.01) was found after therapy (Figs. 3b and 4a, b).

All 10 patients with borderline arteriosclerosis disease had improved laboratory findings after treatment such as higher HDL and lower LDL and triglyceride levels. Of the 44 patients with available normal ABI values (0.9 < ABI < 1.4) before treatment at the same time as CAVI, 43 (97.7%) remained normal after treatment. In general, post-treatment values were considerably decreased compared with pre-treatment values.

## Discussion

n the present study, we retrospectively assessed the safety and effectiveness of autologous Ad-MSC administration in a cohort of patients with arteriosclerosis. Arteriosclerotic diseases comprising ischemic heart disease and cerebrovascular disease are important medical issues closely comparable to malignant neoplasms [1]. In addition, arteriosclerosis is the root of age-related diseases [4].

Recent development of Ad-MSC has provided new opportunity for treatment of arteriosclerosis that could have not been achieved by existing methods and could lead to the development of new treatment concepts. Ischemic heart disease and cerebrovascular disease caused by arteriosclerosis are not only life-threatening, but also cause various reperfusion aftermaths and complications such as impaired motor skills and cognitive dysfunction even after being rescued [1]. As a result, the quality of life and ADL will be reduced, and long-term high-cost medical care will be enforced. In addition, it is necessary to reduce soaring medical costs, prolong the life expectancy of the healthy population, and increase opportunities for elderly people to participate in society. Medical and social responses are urgently needed. Furthermore, the development of therapeutic medicine for arteriosclerosis is directly linked to the development of anti-aging medicine and preemptive medicine. For the above reasons, it can be said that arteriosclerosis is an important issue that medical care should address. MSC are multipotent somatic stem cells present in fat, bone marrow, umbilical cord, and the like in a living body. MSC can differentiate into various mesodermal cells, for example, osteoblasts, chondrocytes, and adipose cells. Because of their pluripotency, MSC are expected to be applied to early regenerative medicine for bone, joint, muscle, liver, kidney, heart, blood vessels, central nervous system, and pancreas. In addition to supplementing and replacing damaged tissues, MSC have various useful functions in vivo by producing various cytokines and extracellular matrices. In particular, studies indicate various immunosuppressive abilities of MSC [26].

The mechanism(s) of beneficial action of MSC has been attributed to a variety of factors. Various studies have shown that MSC exert remarkable beneficial effects, which could make them appropriate for use in treatment of several diseases including arteriosclerosis. These effects include but are not limited to the production of mediators which can reduce inflammation, the potential of MSC to migrate to sites of arterial injury, and their ability to respond based on the extent of the tissue injury. As stated by Mahdavi Gorabi et al. (2019), the migration of MSC is modulated by mediators produced and released by other MSC which affect several receptors and signaling pathways, such as growth factor receptors, G-protein coupled receptor (GPCR), vascular endothelial growth factor/vascular endothelial growth factor receptor (VEGF/VEGFR), stem cell factor-tyrosine kinase receptor (SCF-c-Kit), stromal cell-derived factor-1 (SDF-1)/CXC chemokine receptor-4 (CXCR4), hepatocyte growth factor (HGF/c-Met), platelet-derived growth factor/platelet-derived growth factor receptor (PDGF/PDGFR), monocyte chemoattractant protein-1/CC chemokine receptor 2 (MCP-1/CCR2), and high mobility group box 1/receptor of advanced glycation end products (HMGB1/RAGE) [27].

In addition to multilineage differentiation, MSC can promote tissue regeneration/repair through other mechanisms including paracrine activity by secretion of proteins/peptides and hormones, transferring mitochondria through tunneling nanotubes or microvesicles, and transferring exosomes or microvesicles containing RNA and other molecules [28]. The immunomodulatory effect of MSC treatment on cholesterol metabolism has been demonstrated in mice, by which serum cholesterol levels considerably reduced due to a reduction in very-low-density lipoprotein levels [29]. Allogeneic MSC was shown to restore endothelial function in human heart failure by proliferation of functional endothelial progenitor cells and improvement in vascular reactivity [30]. A long-term follow-up study of intravenous autologous MSC transplantation in patients with ischemic stroke showed functional recovery and lower mortality rate more frequently in the MSC-treated group than in the control group [16]. MSC also have powerful immunomodulatory effects, which include inhibition of cell growth and function of T cells, B cells, and natural killer cells [31]. MSC also secrete various factors that support cell survival, including growth factors, cytokines, and extracellular matrix (ECM) which can promote progenitor cell self-renewal, stimulate angiogenesis, and reduce apoptosis and/or inflammation [28].

On the other hand, conditioned medium or supernatant from culture of MSC (MSC-CM) showed various biological effects after administration. Both extracellular vesicles including exosomes and soluble component of MSC-CM could promote tissue regeneration, inhibit abnormal immune response, and induce angiogenesis in ischemic tissues [32]. Therefore, MSC-CM showed immunoregulatory, angiomodulatory, and anti-apoptotic effects that resulted in enhanced tissue repair and regeneration [33]. Some studies also suggested that MSC-CM may be more effective than MSC in tissue repair [31]. Taken together, these findings suggest that either MSC or MSC-CM can be a good candidate for cell therapy.

Inflammation and immune regulation play a pivotal role in the development of arteriosclerosis and atherosclerotic plaque formation [34, 35] and new therapies to attenuate inflammatory response have been advocated to prevent atherosclerosis [36]. Several clinical trials evaluating the therapeutic potential of MSC from different origins via modulation of inflammation are in progress focusing on the safety and efficacy of such cells to treat and/or alleviate coronary artery disease [27]. It is expected that the upcoming results contribute to development of new therapeutic concepts for arteriosclerosis. Because of the strong anti-inflammatory and immune regulatory function of MSC, as well as their effect on renewing endothelial function [30, 37, 38], we considered that Ad-MSC administration is safe and may be useful in the treatment and prevention of arteriosclerosis. Additionally, Ad-MSC could serve as an effective adjuvant to the standard statin therapy of arteriosclerosis in order to accelerate improvement.

Intravenous route of administration of MSC is commonly used and most MSC are trapped in the lungs and remain transiently there. MSC also distribute in other organs including the liver, spleen, bone marrow, thymus, kidney, and skin [39]. Although the nature of MSC engraftment is said to be transient, it exerts powerful effects as revealed based on the outcome [40]. Direct injection of MSC into the target site ensures precise localization of the MSC. Overall, using different MSC delivery routes may have some effect on their distribution, but rarely on long-term engraftment of the cells, or on clinical outcome [40]. In our series of patients with arteriosclerosis, intravenous route of MSC administration allowed systemic delivery of MSC to vascular endothelial cells which was highly desirable and found to be effective.

In our study, treatment with Ad-MSC significantly improved HDL, LDL, and RLP cholesterol levels. There was no any adverse effect or toxicity in relation to the treatment. This could be attributed to the fact that we had used a proprietary animal-origin-free (AOF) medium for culturing the Ad-MSC which prohibited both adverse immunoreactions and transfer of viral and infectious agents. Currently, it is believed that under certain conditions, circulatory monocytes are recruited to the vascular intimal layer and begin taking up circulatory modified LDL to form macrophage foam cells which are the source of atheroma or plaque formation. The disease mechanism(s) is elicited by LDL and in addition involves apoptosis and necrosis, smooth muscle cell (SMC) proliferation and matrix synthesis, calcification, angiogenesis, arterial remodeling, fibrous cap rupture, thrombosis, and eventually leading to cardiovascular diseases [35, 41, 42]. Pharmacologic therapy of atherosclerosis includes lipid-lowering drugs such as statins that is most effective in reducing the risk of cardiovascular disease [41, 43]. However, several clinical studies have indicated that the treatment with statins still is insufficient and about two thirds of patients continue to be at risk of cardiovascular disease [44–46]. Furthermore, many patients do not stand to continue a long-term statin therapy to maintain an optimal LDL level [43]. Thus, additional modes of therapy for prevention of cardiovascular disease are warranted. In particular, effective lipid-lowering strategies to inhibit macrophage foam cell formation would be highly important. Currently, studies using mesenchymal stem cells for treatment of arteriosclerosis in animal models are ongoing. One animal model of atherosclerosis includes an apolipoprotein E knockout mouse (ApoE-KO mouse). Apolipoprotein E is one of the major apolipoproteins that constitute lipoproteins such as VLDL and HDL, and it is known that deficiency or abnormality causes arterial stiffness and accumulation of cholesterol in blood. In 2015, Wang et al. reported that administration of bone marrow-derived mesenchymal stem cells to ApoE-KO mice improved arteriosclerosis [47]. Li et al. reported that tail-vein administration of skin-derived mesenchymal stem cells to ApoE-KO mice improved arteriosclerosis [48]. These were reports that mesenchymal stem cells regulate macrophages involved in arterial plaque formation. Wang et al. reported that administration of bone marrow-derived mesenchymal stem cells to the ear vein improved arteriosclerosis using a rabbit atherosclerosis model [49]. It has been reported that inflammation is involved in the formation of arterial plaque [50]. It was suggested that the inflammatory effect might have improved arteriosclerosis. In clinical studies, Gupta et al. in 2013 showed the efficacy of intramuscular administration of bone marrow-derived mesenchymal stem cells against lower limb arterial ischemia [51]. Furthermore, in a recent report, we investigated the safety and efficacy of intravenous autologous Ad-MSC in a patient with acute myocardial infarction. We found that the administration of Ad-MSC was safe and was associated with improved recovery of the left ventricular function, electrocardiographic findings, and serum BNP level. It was concluded that Ad-MSC may be considered to be one of future therapeutic agents for diseases that cannot be cured by conventional therapeutic methods [52].

In the present study, a significant decrease was found in the IMT values after therapy (*P* < 0.01). Previous studies did not address the effect of stem cell therapy on IMT, which does not allow a comparative assessment. Taken together, our results suggest that at least the administration of Ad-MSC had prevented the progression of arteriosclerosis which is an important issue in the management of this disease.

Studies on tracking MSC in vivo using animal models showed that a small proportion of the cells home and persists in the target sites and most of the cells are not detectable after 7~14 days post transplantation. MSC were almost undetectable in all tissues by 21 days after injection [53]. Nevertheless, the effects of MSC transplantation appear to persist for a longer time as seen in patients with osteoarthritis and 30 months follow-up [54]. We plan to follow-up our patients and collect more information in this regard.

Some limitations of our study should be mentioned of which the time periods of data between before and after AD-MSC administrations and the frequency of administrations varied from patient to patient. Patient cooperation was essential for follow-up testing after stem cell administration, as both consultation and examinations required a visit to our clinic.

Our results of statistically significant improvement in CAVI levels indicate that AD-MSC may have prevented the development of atherosclerosis. CAVI is a well-known indicator of arterial stiffness and is widely used because of its ease of examination. CAVI can be measured simultaneously with the measurement of ABI which is the ratio of the blood pressure at the ankle to the blood pressure in the arm and both are test methods with little burden for patients. The vast majority of patients with normal ABI values before treatment at the same time as CAVI remained normal after treatment.

## Conclusion

In conclusion, based on the results of this study, Ad-MSC administration in patients developing arteriosclerosis was safe and effective. The patients demonstrated improvements in clinical manifestations after therapy. No toxicity or serious adverse effects were observed. Thus, Ad-MSC may provide physicians with an adjunct therapy for patients with arteriosclerosis. Furthermore, the results of this study encourage subsequent randomized controlled trials to confirm the therapeutic application of Ad-MSC in arteriosclerosis.

## Data Availability

The processed data supporting the findings of this retrospective study are available from the corresponding author upon request.

## Abbreviations

Ad-MSC: Adipose-derived mesenchymal stem cells
IMT: Intimal-media thickness
CAVI: Cardio-ankle vascular index
ABI: Ankle-brachial index
HDL: High-density lipoprotein
LDL: Low-density lipoprotein
RLP: Remnant-like particle
MSC: Mesenchymal stem cells
CM: Conditioned medium
IL-1α: Interleukin-1α
IL-1β: Interleukin-1β
IL-6: Interleukin 6
TNF-α: Tumor necrosis factor-α
CSF: Cerebrospinal fluid
QOL: Quality of life
ADL: Activities of daily living
PWV: Pulse wave velocity
HIV: Human immunodeficiency virus
HCV: Hepatitis C virus
CLIA: Chemiluminescence immunoassay
HB: Hepatitis B
HTLV-I: Human T-lymphotropic virus
RPR: Rapid plasma regain
TPHA: Treponema pallidum particle agglutination assay
CF: Complement fixation
PA: Passive agglutination
AOF: Animal-origin-free
SMC: Smooth muscle cell
VLDL: Very-low-density lipoprotein
BNP: Brain natriuretic peptide

## References

[1] Benjamin EJ, Muntner P, Alonso A (2019). Heart disease and stroke statistics-2019 update: a report from the American Heart Association. Circulation.

[2] Ohta H, Ohki T, Kanaoka Y (2016). Hybrid approach to complex arch and thoracoabdominal aneurysms in a case of high-risk patient. Clin Surg.

[3] Ohta H, Ohki T, Kanaoka Y (2017). Pitfalls of invasive blood pressure monitoring using the caudal ventral artery in rats. Sci Rep.

[4] Kunz J (2000). Initial lesions of vascular aging disease (arteriosclerosis). Gerontology.

[5] Chobanian AV (1992). Pathophysiology of atherosclerosis. Am J Cardiol.

[6] Egusa G, Watanabe H, Ohshita R (2002). Influence of the extent of westernization of lifestyle on the progression of preclinical atherosclerosis in Japanese subjects. J Atheroscler Thromb.

[7] Watanabe H, Yamane K, Fujikawa R, Okubo M, Egusa G, Kohno N (2003). Westernization of lifestyle markedly increases carotid intima-media wall thickness (IMT) in Japanese people. Atherosclerosis..

[8] O'Leary DH, Polak JF, Kronmal RA, Manolio TA, Burke GL, Wolfson SK (1999). Carotid-artery intima and media thickness as a risk factor for myocardial infarction and stroke in older adults. Cardiovascular Health Study Collaborative Research Group. Engl J Med.

[9] Nathan DM, Lachin J, Cleary P, Orchard T, Brillon DJ, Backlund JY, O'Leary DH, Genuth S (2003). Diabetes control and complications trial, epidemiology of diabetes interventions and complications research group. Intensive diabetes therapy and carotid intima-media thickness in type 1 diabetes mellitus. N Engl J Med.

[10] Ono K, Tsuchida A, Kawai H, Matsuo H, Wakamatsu R (2003). Ankle-brachial blood pressure index predicts all-cause and cardiovascular mortality in hemodialysis patients. J Am Soc Nephrol.

[11] Namba T, Masaki N, Takase B, Adachi T (2019). Arterial stiffness assessed by cardio-ankle vascular index. Int J Mol Sci.

[12] Gimble JM, Katz AJ, Bunnell BA (2007). Adipose-derived stem cells for regenerative medicine. Circ Res.

[13] Vonk LA, de Windt TS, Slaper-Cortenbach IC, Saris DB (2015). Autologous, allogeneic, induced pluripotent stem cell or a combination stem cell therapy? Where are we headed in cartilage repair and why: a concise review. Stem Cell Res Ther.

[14] Bajek A, Gurtowska N, Olkowska J, Kazmierski L, Maj M, Drewa T (2016). Adipose-derived stem cells as a tool in cell-based therapies. Arch Immunol Ther Exp.

[15] Hirano A, Sano M, Urushihata N, Tanemura H, Oki K, Suzaki E (2018). Assessment of safety and feasibility of human allogeneic adipose-derived mesenchymal stem cells in a pediatric patient. Ped Res.

[16] Lee JS, Hong JM, Moon GJ, Lee PH, Ahn YH, Bang OY, STARTING collaborators (2010). A long-term follow-up study of intravenous autologous mesenchymal stem cell transplantation in patients with ischemic stroke. Stem Cells.

[17] Seetharaman R, Mahmood A, Kshatriya P, Patel D, Srivastava A. "Mesenchymal Stem Cell Conditioned Media Ameliorate Psoriasis Vulgaris: A Case Study", Case Reports in Dermatological Medicine. 2019;2019(Article ID 8309103):5. 10.1155/2019/8309103. 10.1155/2019/8309103 PMC6521531 31186972

[18] Ahn SY, Chang YS, Sung DK (2014). Mesenchymal stem cells prevent hydrocephalus after severe intraventricular hemorrhage. Stroke.

[19] MacFarlane RJ, Graham SM, Davies PS (2013). Anti-inflammatory role and immunomodulation of mesechymal stem cells in systemic joint diseases: potential for treatment. Expert Opin Ther Targets.

[20] Ramasamy R, Fazekasova H, Lam EW, Soeiro I, Lombardi G, Dazzi F (2007). Mesenchymal stem cells inhibit dendritic cell differentiation and function by preventing entry into the cell cycle. Transplantation.

[21] Mahmoudifar N, Doran PM (2015). Mesenchymal stem cells derived from human adipose tissue. Methods Mol Biol.

[22] Francis MP, Sachs PC, Lynne W, Elmore LW, Holt SE (2010). Isolating adipose-derived mesenchymal stem cells from lipoaspirate blood and saline fraction. Organogenesis.

[23] Stocchero IN, Stocchero GF, Illouz Y-G, Sterodimas A (2011). Isolation of stem cells from human adipose tissue: technique, problems, and pearls. Adipose stem cells and regenerative medicine.

[24] Vériter S, André W, Aouassar N, Poirel HA, Lafosse A, Docquier P-L (2015). Human adipose-derived mesenchymal stem cells in cell therapy: safety and feasibility in different “hospital exemption” clinical applications. Plos One.

[25] Aso K, Tsuruhara A, Takagaki K, Oki K, Ota M, Nose Y, Tanemura H, Urushihata N, Sasanuma J, Sano M, Hirano A, Aso R, McGhee R, Fujihashi K (2016). Adipose-derived mesenchymal stem cells restore impaired mucosal immune responses in aged mice. Plos One.

[26] Newman RE, Yoo D, LeRoux MA, Danilkovitch-Miagkova A. Treatment of inflammatory diseases with mesenchymal stem cells. Inflamm Allergy Drug Targets. 2009;8(2):110–23. 10.2174/187152809788462635. 10.2174/187152809788462635 19530993

[27] Mahdavi Gorabi A, Banach M, Reiner Z, Pirro M, Hajighasemi S, Johnston TP, Sahebkar A (2019). The role of mesenchymal stem cells in atherosclerosis: prospects for therapy via the modulation of inflammatory milieu. J Clin Med.

[28] Spees JL, Lee RH, Gregory CA (2016). Mechanisms of mesenchymal stem/stromal cell function. Stem Cell Res Ther.

[29] Frodermann V, Van Duijn J, Van Pel M, Van Santbrink PJ, Bot I, Kuiper J, De Jager SC (2015). Mesenchymal stem cells reduce murine atherosclerosis development. Sci Rep.

[30] Premer C, Blum A, Bellio MA, Schulman IH, Hurwitz BE, Parker M, Dermarkarian CR, DiFede DL, Balkan W, Khan A, Hare JM (2015). Allogeneic mesenchymal stem cells restore endothelial function in heart failure by stimulating endothelial progenitor cells. EBioMedicine.

[31] Gnecchi M, Danieli P, Malpasso G, Ciuffreda MC (2016). Paracrine mechanisms of mesenchymal stem cells in tissue repair. Methods Mol Biol.

[32] Vilaça-Faria H, Salgado AJ, Teixeira FG (2019). Mesenchymal stem cells-derived exosomes: a new possible therapeutic strategy for Parkinson’s disease?. Cells.

[33] Harrell CR, Fellabaum C, Jovicic N, Djonov V, Arsenijevic N, Volarevic V (2019). Molecular mechanisms responsible for therapeutic potential of mesenchymal stem cell-derived secretome. Cells..

[34] de Jager SC, Pasterkamp G (2013). Crosstalk of lipids and inflammation in atherosclerosis: the PRO of PGRN?. Cardiovasc Res.

[35] Frostegard J (2013). Immunity, atherosclerosis and cardiovascular disease. BMC Med.

[36] Khan R, Spagnoli V, Tardif JC, L’Allier PL (2015). Novel anti-inflammatory therapy for the treatment of atherosclerosis. Atherosclerosis.

[37] Lin YL, Yet SF, Hsu YT, Wang GJ, Hung SC (2015). Mesenchymal stem cells ameliorate atherosclerotic lesions via restoring endothelial function. Stem Cells Transl Med.

[38] Ratushnyy A, Ezdakova M, Yakubets D, Buravkova L (2018). Angiogenic activity of human adipose-derived mesenchymal stem cells under simulated microgravity. Stem Cells Dev.

[39] Barbash IM, Chouraqui P, Baron J, Feinberg MS, Etzion S, Tessone A, Miller L, Guetta E, Zipori D, Kedes LH, Kloner RA, Leor J (2003). Systemic delivery of bone marrow-derived mesenchymal stem cells to the infarcted myocardium: feasibility, cell migration, and body distribution. Circulation..

[40] Kurtz A (2008). Mesenchymal stem cell delivery routes and fate. Int J Stem Cells.

[41] Wigren M, Nilsson J, Kolbus D (2012). Lymphocytes in atherosclerosis. Clin Chim Acta.

[42] Major AS, Harrison DG (2011). What fans the fire: insights into mechanisms of inflammation in atherosclerosis and diabetes mellitus. Circulation.

[43] Shapiro MD, Fazio S (2016). From lipids to inflammation. Circ Res.

[44] Hague W, Forder P, Simes J, Hunt D, Tonkin A (2003). Effect of pravastatin on cardiovascular events and mortality in 1516 women with coronary heart disease: results from the long-term intervention with pravastatin in ischemic disease (LIPID) study. Am Heart J.

[45] Libby P (2015). The forgotten majority: unfinished business in cadiovascular risk reduction. J Am Coll Cardiol.

[46] Serban MC, Banach M, Mikhailidis DP (2016). Clinical implications of the IMPROVE-IT trial in the light of current and future lipid-lowering treatment options. Expert Opin Pharmacother.

[47] Wang ZX, Wang CQ, Li XY, Feng GK, Zhu HL, Ding Y, Jiang XJ (2015). Mesenchymal stem cells alleviate atherosclerosis by elevating number and function of CD4(+)CD25(+)FOXP3(+) regulatory T-cells and inhibiting macrophage foam cell formation. Mol Cell Biochem.

[48] Li Q, Sun W, Wang X, Zhang K, Xi W, Gao P (2015). Skin-derived mesenchymal stem cells alleviate atherosclerosis via modulating macrophage function. Stem Cells Transl Med.

[49] Wang SS, Hu SW, Zhang QH, Xia AX, Jiang ZX, Chen XM (2015). Mesenchymal stem cells stabilize atherosclerotic vulnerable plaque by anti-inflammatory properties. PLoS One.

[50] Libby P (2002). Inflammation in Atherosclerosis. Nature.

[51] Gupta PK, Chullikana A, Desai RPS, Das A, Gottipamula S, Krishnamurthy S, Anthony N, Pherwani A, Majumdar AS (2013). A double blind randomized placebo controlled phase I/II study assessing the safety and efficacy of allogeneic bone marrow derived mesenchymal stem cell in critical limb ischemia. J Transl Med.

[52] Mishima M, Liu X (2019). Evaluation of autologous adipose-derived mesenchymal stem cell therapy in a patient with acute ischemic cardiomyopathy. Am J Exp Clin Res.

[53] Casiraghi F, Azzollini N, Cassis P, Imberti B, Morigi M, Cugini D, Cavinato RA, Todeschini M, Solini S, Sonzogni A, Perico N, Remuzzi G, Noris M (2008). Pre-transplant infusion of mesenchymal stem cells prolongs the survival of a semi-allogeneic heart transplant through the generation of regulatory T cells. J Immunol.

[54] Emadedin M, Ghorbani Liastani M, Fazeli R, Mohseni F, Moghadasali R, Mardpour S, Hosseini SE, Niknejadi M, Moeininia F, Aghahossein Fanni A, Baghban Eslaminejhad R, Vosough Dizaji A, Labibzadeh N, Mirazimi Bafghi A, Baharvand H, Aghdami N (2015). Long-term follow-up of intra-articular injection of autologous mesenchymal stem cells in patients with knee, ankle, or hip osteoarthritis. Arch Iran Med.

